# Known unknowns: building an ethics of uncertainty into genomic medicine

**DOI:** 10.1186/s12920-016-0219-0

**Published:** 2016-09-01

**Authors:** Ainsley J. Newson, Samantha J. Leonard, Alison Hall, Clara L. Gaff

**Affiliations:** 1 Centre for Values, Ethics and the Law in Medicine, Sydney School of Public Health, University of Sydney, Level 1, Medical Foundation Building K25, 92-94, Parramatta Road, Camperdown, NSW 2006 Australia; 2 Inserm, Unite 1027, University Toulouse III, Paul Sabatier, France; 3 Service de Genetique Medicale CHU Toulouse, Toulouse, France; 4 PHG Foundation, Cambridge, UK; 5 Melbourne Genomics Health Alliance, Melbourne, Australia; 6 Departments of Paediatrics and Medicine, University of Melbourne, Melbourne, Australia

**Keywords:** Ethics, Uncertainty, Genomics, Clinical genomics, Massively parallel sequencing, Genome sequencing, Genomic testing, Genetic counseling, Rare diseases, Variants of uncertain significance

## Abstract

**Background:**

Genomic testing has reached the point where, technically at least, it can be cheaper to undertake panel-, exome- or whole genome testing than it is to sequence a single gene. An attribute of these approaches is that information gleaned will often have uncertain significance. In addition to the challenges this presents for pre-test counseling and informed consent, a further consideration emerges over how - ethically - we should conceive of and respond to this uncertainty. To date, the ethical aspects of uncertainty in genomics have remained under-explored.

**Discussion:**

In this paper, we draft a conceptual and ethical response to the question of how to conceive of and respond to uncertainty in genomic medicine. After introducing the problem, we articulate a concept of ‘genomic uncertainty’. Drawing on this, together with exemplar clinical cases and related empirical literature, we then critique the presumption that uncertainty is always problematic and something to be avoided, or eradicated. We conclude by outlining an ‘ethics of genomic uncertainty’; describing how we might handle uncertainty in genomic medicine. This involves fostering resilience, welfare, autonomy and solidarity.

**Conclusions:**

Uncertainty will be an inherent aspect of clinical practice in genomics for some time to come. Genomic testing should not be offered with the explicit aim to reduce uncertainty. Rather, uncertainty should be appraised, adapted to and communicated about as part of the process of offering and providing genomic information.

## Background

Genomic sequencing is already aiding diagnosis in many individuals and families who until now have had unknown or unexplainable causes for the conditions they live with [1]. Its use is increasing and there is enthusiasm for receiving genomic information [2–4]. However while genetic testing has always carried with it the prospect that uncertain information would arise, [5, 6] the increased scale of genomic testing methods such as panel-, exome- or whole genome sequencing (and the information they could give rise to) means that results with uncertain significance, uncertain prognostic indicators or a meaning that changes over time are now more prevalent [7–11]. Uncertainty is, and will continue to be, inherent to genomic medicine [12, 13].

Accounting for uncertainty in genomics carries with it clinical and counseling dimensions, such as appropriate preparation for uncertainty and good communication [6, 8, 14] when information is uncertain. Empirical studies are already providing useful data on how uncertainty is approached by practitioners in genomics research and clinical practice; as well as the impact it has on those who receive information that is uncertain [8, 10, 12, 15, 16]. However we contend there is a negative presumption underlying much current management of uncertainty in genomics. Uncertainty tends to be framed as inherently pejorative or harmful and something to be avoided or eradicated. This also reflects more general views on uncertainty in academic writing [17, 18], such as the assertion that “doctors… are learning to be afraid of uncertainty [19].”

In this paper, rather than looking at the actual impact of uncertainty, we focus on its *normative status* within genomic medicine. We ask: how should we conceive of and respond to ‘uncertainty’ in genomic medicine? Our answer is that while we do not wish to underplay the impact that uncertainty can have in genomic testing, a presumption that uncertainty is necessarily problematic or needs to be eradicated should be rejected. We develop a conceptualization of ‘genomic uncertainty’ and call for its systematic and explicit incorporation into clinical genomic practice^1^; a position that emphasizes ethically relevant concepts such as resilience, welfare, autonomy and solidarity. Additionally, while there are some forms of uncertainty in genomics that it is desirable to try to reduce, others can be framed as a source of options and hope for the future [6].

## Discussion

### Conceptualizing ‘uncertainty’ and ‘genomic uncertainty’

Before discussing how we might account for genomic uncertainty, we need to consider what is actually under discussion when we speak of ‘uncertainty’. Uncertainty is a cluster concept that has many different - but associated - meanings [14, 18, 20]. Broadly conceived, uncertainty can be considered as a *state of having imperfect or unknown information*.^2^

This imperfect or unknown information can arise in genomics in at least two contexts: (i) clinical diagnosis and (ii) clinical prognosis and/or management. It is also a property that can be recognized subjectively (experientially) by an individual provider or recipient of the information, and/or viewed as an objective property of the test or information itself.^3^

While a full survey of uncertainty scholarship is beyond the scope of this paper, we have drawn on two formulations to inform our conception of genomic uncertainty: Han et al’s *sources* of uncertainty [21] and Babrow et al’s *forms* of uncertainty [14].

Han et al. [21] describe uncertainty in terms of its *sources*: probability; ambiguity and complexity. These are properties of information that make it uncertain. These sources are intrinsic to much genomic medicine, in that uncertainty is characteristic of the information communicated as part of genomic testing. Thus, we can apply Han et al’s taxonomy as follows:

- Probability uncertainty occurs where there is indeterminacy of future outcomes. Below, we discuss the case of Jennifer (Case 1), who receives a result that suggests she has a probability of developing a highly invasive cancer. But it is uncertain whether she will actually develop it.
- Ambiguity uncertainty arises when the information or evidence is imprecise, where there is conflicting opinion or where information is not known. Aisha (Case 2 below) is experiencing ambiguity uncertainty as we are unable to tell her the significance of the 400 kb microdeletion identified in her fetus.
- Complexity uncertainty arises when there are features of the available information that make it hard to understand. Factors such as epigenetic effects, gene-gene or gene-environment interactions, pleiotropy and unknown penetrance in previously untested clinical populations mean that much genomic information will have this property. Darnel’s case (Case 3) exemplifies complexity uncertainty.

Babrow et al. caution that definitions of uncertainty can be criticized for treating uncertainty as monolithic [22]. They suggest five forms of uncertainty, which use similar terminology to that of Han et al. but more explicitly account for how an individual will experience uncertainty. We have applied these forms to genomic testing as follows:

1. Inherent uncertainty: The test process may give rise to uncertainty. There may be multiple causes of a particular condition, not all of which might be identifiable with current testing. The condition may interact with others, affecting interpretation of results. In advance, we cannot always be certain about what results may arise or what they might mean.^4^
2. Informational uncertainty: Uncertainty can arise from the sufficiency, clarity, accuracy, completeness, ambiguity, volume, reliability, consistency and validity of information arising from genomic testing.
3. Views on uncertainty: This form of uncertainty describes recipients’ views on the likelihood (probability) of a certain test outcome.^5^ A recipient may have a set probability in mind, or believe in a range of probabilities that could arise from genomic testing.
4. Structuring of information: Uncertainty can also arise from how recipients might structure, order or integrate the information they receive in genomic testing with respect to existing beliefs, attitudes and values.
5. Personal views about knowledge: Uncertainty can be interpreted differently depending on variations in individual attitudes towards ‘knowledge’. For example, there will be different views on whether uncertainty is tolerated, rejected or perhaps even required in the pursuit of knowledge through genomic testing.

Babrow et al’s first and second forms of uncertainty overlap with Han et al’s three sources of uncertainty. In their third, fourth and fifth forms, Babrow et al. seem to more explicitly recognize the experiential or subjective properties of uncertainty. This is ethically relevant as these may be more easily addressed by clinical encounters in genomics. Han et al., in contrast, seem to focus primarily on properties relating to the test itself.

We build on these conceptualizations of uncertainty to suggest the following definition of genomic uncertainty:

Genomic uncertainty: is a status quo that arises when information that is obtained from genomic testing is imperfect or unknown, leading to uncertainty in clinical diagnosis or management. Genomic uncertainty can arise from the probabilistic, ambiguous or complexity uncertainty inherent to the information arising from testing, or from the provider’s or recipient’s views on and uses of it.

### Uncertainties relevant to genomics: exemplar clinical contexts

Uncertainty can arise in any form of genomic testing. However some indicative clinical case scenarios can assist in illustrating how and when uncertainty can arise. Consider the cases of Aisha, Jennifer and Darnel. These scenarios all meet the definition of genomic uncertainty as described above.

#### Case 1: Jennifer’s panel test [23]

Jennifer is 39 years old and is fit and well. However, she is concerned about her mutation status for BRCA1 and BRCA2 as she has a relevant family history. She therefore seeks testing. During the consent process for this test, Jennifer is offered panel testing for 20 other cancer-related genes. She agrees to receive this information. Test results indicate a mutation in a gene responsible for stomach cancer (but no BRCA1 or BRCA2 mutation). One intervention to reduce this particular risk is a surgical procedure that involves removing large portions of the stomach. However, Jennifer has no family history of this cancer and is keen to avoid unnecessary surgery. “It’s so up in the air”, she says.

Jennifer’s test result has uncertainty for her ongoing management. The mutation has a known etiology, but her prognosis in the absence of any family history is uncertain. The scenario would be indicative of at least Han at al’s probability and complexity sources of uncertainty and Babrow et al’s second (and possibly fourth) conceptions; uncertainty arising from the test and structuring of information.

#### Case 2: Aisha’s test during pregnancy [24]

Aisha is a 29 year-old primigravid woman, who is 21 weeks pregnant. A recent ultrasound indicated the presence of some anomalies. As a result, Aisha is offered microarray testing, with counseling. After careful consideration and deliberation, Aisha and her partner decide to proceed with testing. Results indicate a novel, de novo 400 kb microdeletion that contains four genes. None of these genes are known to be associated with human disease.

Aisha’s test result suggests uncertainty in both diagnosis and management. Aisha’s tolerance for uncertainty will inform how her pregnancy progresses from this point. It would meet at least Han et al’s description of ambiguity uncertainty, and may also exemplify Babrow et al’s inherent and informational forms, given that a phenotype that correlates with this microdeletion cannot be predicted.

#### Case 3: explaining Darnel’s cancer

Darnel is 47 years old and has experienced two episodes of bowel cancer consistent with HNPCC. He has no relevant family history, but given his cancer’s recurrence and clinical presentation, a genetic link was suspected. A panel test filtered to the genes associated with HNPCC found no mutation.

Darnel’s result also indicates uncertainty in diagnosis and management. It is an example of Han et al’s complexity uncertainty (perhaps a gene-environment interaction), and Babrow et al’s inherent uncertainty.

Aisha, Jennifer and Darnel have received uncertain information following genomic testing. While we don’t know enough from these descriptions to consider in detail Babrow et al’s experiential forms of uncertainty (their third, fourth and fifth forms), it is reasonable to suggest that these individuals would have sought testing to obtain information and presumably reduce uncertainty [25–27]. Their cases highlight several considerations such as the support recipients may need to both adjust to living with uncertainty and make subsequent decisions in light of the information they’ve received.

Supporting uncertainty through processes such as genetic counseling is important, but is not the focus of this paper. Instead, we question whether uncertainty should always be treated and presented as something to be ‘avoided,’ [12] and the extent to which certain framings might detract from more beneficial responses such as encouraging resilience, promoting welfare and autonomy, and fostering solidarity. We go on to discuss that uncertainty should be appraised as part of the process of undergoing any genomic test.

### What do we know about genomic uncertainty?

Empirical research into expectations and experiences of uncertainty in genomics is now emerging, and measures of responses to uncertainty are being developed [8]. Previous studies that looked at uncertainty in *genetic* testing suggest that testing is sought to reduce uncertainty [25] or to end a diagnostic odyssey, [27] but that those who do not achieve certainty are often nevertheless resilient [28]. In a genomics context, ‘early adopters’ of genomic testing tend to be comfortable with uncertainty, [29] although if an individual perceives that genomic testing results are going to be ambiguous they are more likely to show reduced intentions to receive them or share them with family [10, 30].^6^

Biesecker *et al*. [12] found that prior attitudes to genomics (whether a person was generally more optimistic or pessimistic) were relevant to individual attitudes towards uncertainty; a point Babrow et al. [14, 22] also recognize more generally. Several participants in Biesecker et al’s study reported that uncertainty was normal and to be expected given the relatively recent emergence of genomic testing. Concepts of opportunity and optimism regarding the future also emerged. However, other participants expressed disappointment and feelings of perplexity and anxiety at having received uncertain information, and felt less hopeful. We discuss this study and its recommendations further below.

### Is uncertainty ethically relevant?

As a stand-alone concept, ‘uncertainty’ may not be inherently ‘ethical’ in the way that other concepts often cited in bioethics (such as ‘autonomy’ or ‘virtue’) are. Uncertainty is, however, ethically relevant. For example, there are both conceptual and empirical ties between notions of uncertainty and hope [6, 13, 22, 31–33]. But experiences of uncertainty might also mean that recipients of uncertain genomic information may experience stress, a reduced sense of coherence and loss of control.

Bauman posits a theoretical approach to ethics that is guided by a ‘principle of uncertainty’ [34]. He claims that uncertainty is a permanent aspect of our lives and cannot be resolved through activities such as taking advice or copying others. The sources and forms of uncertainty in genomics can be said to fit this description. Bauman then claims that any moral life is one that involves continuous uncertainty, reflecting an epistemological view that would fit into Babrow et al’s fifth form (personal views on knowledge). As such, Bauman claims that any ethical theory needs to incorporate uncertainty.

Heath has also used work by Nussbaum and Toulmin to claim that uncertainty is inherent to leading a life worth living [19]. Our inability to control every aspect of our lives and the unpredictable nature of our futures are important aspects of living a rich life. Aiming for precise prediction may set us up for a kind of genomic healthcare that is doomed to fail. Instead, we need to encompass a model of care that builds in a recognition and acceptance of uncertainty and all that this brings with it.

Han has written about the possible detrimental impact of uncertainty on autonomy [20]. He claims that people need and deserve uncertain information, as it can be autonomy-promoting. However, the sources of uncertainty mentioned above (probability, ambiguity and complexity) could also give rise to harms such as anxiety - effectively undermining rather than promoting autonomy.

### Ethics and reducing uncertainty

We have seen so far that uncertainty is prevalent in genomic medicine and that it is something that health professionals working in genomics might want to avoid or eradicate, given its apparent negative framing. Here we suggest that clinical teams working with those who will obtain genomic information like Jennifer, Aisha and Darnel should focus explicitly on the role of uncertainty in genomics during pre-test discussions; directly acknowledging and appraising it rather than merely conceiving of it as problematic or seeking to avoid a negative response to it.

Our position reflects work by Babrow and Kline in the context of breast self-examination [22] and also Skirton and Bylund in genetics [16] Babrow and Kline make two claims. The first relates to the rhetoric that often surrounds a health intervention, namely its use as a means of reducing “unpleasant uncertainty [22].” They observe that breast screening does not necessarily reduce uncertainty over breast cancer risk. The same is true for genomic testing: it will not necessarily resolve uncertainty over disease risk or prognosis and may lead to new forms of uncertainty. This is not to say that breast self-examination or genomic testing should not be promoted or performed; but that the rhetoric of ‘test to reduce uncertainty’ is not appropriate because this is not what testing does.

Babrow and Kline’s second concern is more fundamental. It draws on the premise that we have identified earlier, namely that: “uncertainty can – and should – be eradicated [22].” In both breast self-examination and genomic testing (as well as, no doubt, other health domains) testing tends to be offered as a means of eradicating uncertainty; of getting to ‘an answer’. However, as the above cases illustrate, the testing process itself can either fail to resolve uncertainty or introduce further uncertainty. It is intuitive to want to reduce uncertainty - as certainty may bring with it perceived feelings of control, coherence and reduced stress. However Babrow and Kline critique “the ideology of uncertainty reduction”; expressing concern over its “unreflective promotion.”

We agree that uncertainty should not necessarily be framed as something undesirable that must be eradicated. Rather, discussion of uncertainty should be integrated into the entire genomic testing process in a way so as to promote resilience if it is not resolved. Babrow and Kline term this *coping with uncertainty*, which involves: appraisal, adaptation and complexity in communication [22].

Appraisal involves examining the sources and forms (and relevance [32]) of uncertainty that can arise and then determining their value, disvalue or neutrality. Responses could be to reduce it (if an uncertainty is relevant but negative, and can be reduced) or perhaps to come to terms with it (if it is relevant, neutral but cannot be altered). This may facilitate active coping or resilience in the face of uncertainty, rather than merely tolerating it. It will also facilitate any positive uncertainties to be used adaptively in counseling.

An appraisal process will require genetics professionals and test recipients to work together to identify suitable coping strategies and responses, such as emphasizing continuity of care regardless of uncertainty. In Jennifer’s case (Case 1), for example, the potential uncertainty from the additional genes to be tested for in the panel should have been discussed prior to testing. Jennifer should have had an opportunity to consider potential uncertainty and her attitudes towards it (whether positive, negative or neutral); and to work this into her decision-making.

Adaptation involves active adjustment to the particular kind of uncertainty that has arisen. Babrow and Kline claim that this should involve understanding the particular nature of the uncertainty that has arisen (or may arise) as well as critically appraising the information that has been provided [22]. Aisha’s case (Case 2) illustrates that adaptation in a genomics context will not always be straightforward. It may involve providers having to critically appraise information that has shifting meaning and may be significant in volume and detail. The source and reliability of this information of uncertain significance needs to be clearly explained, and Aisha (and her partner) should be offered appropriate follow-up.

Acknowledging complexity in communication involves understanding communication not as a one-way transaction, but as something more complex. Resolving uncertainty does not just involve providing better information or adopting an approach such as “conveying these uncertainties [12].” Communication needs to carefully negotiate uncertainty, taking into account the appraisal and adaptation that has already taken place [22]. In Darnel’s case (Case 3), this will also involve regular invitations to re-engage with the team who provided testing. This will enable his views on the results to be fed back to the team, noting that his subjective experience of the uncertainty may change over time. It will also allow additional appraisal and adaption.

The practice we advocate here is consistent with predominant definitions of genetic counseling, which includes facilitating adaptation [35]. Skirton and Byland have also described a similar process for managing uncertainty [6]. However, we need to do more to step away from any ideology around uncertainty eradication; and re-frame uncertainty from something that is intuitively negative to something that is appraised (or managed [6]) in a more value-neutral way. Additionally, this discussion illustrates the necessity for genomic testing to be accompanied by structured support for decision-making - an approach that may not easily be sustained by all models of genomic testing, such as direct to consumer tests.

### Towards an ‘ethics of uncertainty’

We have engaged with two claims in this paper: First, that uncertainty is necessarily pejorative; and second, that fostering an accurate understanding of the current state of genomics will reduce adverse responses to uncertainty [12]. We query both of these claims.

On the first claim, we have shown that uncertainty is itself a complex concept; one that will manifest in different ways in genomic testing. Testing can reduce some kinds of uncertainty but it will not reduce all uncertainty and may introduce new uncertainties. Drawing on work from health communication, we have suggested that a structured and supportive integration of uncertainties, and a determination of reactions to them throughout the genomic testing process, will help ensure that uncertainty is constructively incorporated into clinical practice. This means that uncertainties can be characterized in a variety of ways [36]. We should also recognize “… that uncertainty can be a door to hope, an opportunity or challenge, or a threat [14].”

As outlined above, Biesecker at al’s study suggests that personal views will affect how someone responds to uncertainty [12]. In response to their findings, they recommend that in advance of genomic testing, researchers could engage in “assessing and modifying these [participant’s] beliefs, through the provision of an epistemological [knowledge-based] intervention…”, adding that this “…may be a key to enhancing informed choice and mitigating negative responses to the uncertainty [12].” Their discussion also suggests a presumption that uncertain results should always be disclosed. However, we should not assume that potential recipients of genomic information need to have their beliefs modified so as to avoid a negative response to uncertainty. This risks pre-empting judgements about what kinds of uncertainty are appropriate. Rather than attempting to change views, the process of managing uncertainty needs to both attend to the more experiential aspects of uncertainty and engage in appraisal, adaptation and complex communication. We therefore agree with Taber et al. that clinicians should: “focus on addressing responses to perceived ambiguity rather than on reducing perceived ambiguity itself…” [10] We also should not presume that uncertain information (when it does arise) will always be returned to recipients. Rather, this is something to be negotiated as part of the appraisal process.

Building an ethics of uncertainty into genomic medicine can also draw on additional ethical concepts. We suggest these can include promoting resilience, welfare, autonomy and solidarity.

**Resilience** in the face of uncertain genomic information can be said to be the ability of an individual or family to deal effectively with information received. Encouraging resilience will recognize that the uncertainties in genomic testing will impact those tested. The testing process and outcome may be positive, neutral or negative, but it will require some time to process. An approach that builds in resilience will recognize this and will allow for some time to ‘recalibrate’ following testing.

Similarly, promoting **welfare** among those undergoing genomic testing will involve ‘looking out’ for their well-being (that is, internal states such as happiness or satisfaction of preferences) throughout the testing process. The framing and handling of uncertainties may impact welfare and the approaches we have discussed in this paper may assist in welfare promotion. Part of this involves getting the balance right between providing too little or too much space for discussing uncertainty. For example, an outcome harmful to welfare would be one in which an uncertainty discussion resulted in that fact alone meaning an individual eschewing information that was clearly in her best interests to receive.

It could also be claimed that a truly **autonomous** decision to undertake genomic testing – one that demonstrates individual or relational self-governance and critical reflection, drawing on relevant beliefs and values – will also incorporate a constructive and honest appraisal of uncertainty. This involves working in partnership with those undertaking testing; not merely leaving them to make a decision, having been ‘educated’.

Han posits a relationship between access to information and autonomy [20]. However, we should be wary of assuming that all information is good or desired by its potential recipients. Instead, we need to encourage critical reflection on information – its kinds and its volume – and the role that uncertainty plays in this. Seely et al. [18] suggest that: “acknowledging uncertainty does not mean abandoning patients to their autonomy… By acknowledging uncertainty within patient care, the physician-patient relationship can be elevated to one of greater communication and shared decision-making.”

Finally, an approach to uncertainty in genomics can build in **solidarity**. One definition of solidarity involves signifying “shared practices reflecting a collective commitment to carry costs (financial, social, emotional, or otherwise) to assist others [37].” The ‘costs’ in this case will be those arising from genomic uncertainty and its effective appraisal. Recognising solidarity between providers and recipients of genomic information will emphasize mutual support, facilitating those undertaking testing to feel that they are in partnership with those offering it.

## Conclusions

This Debate article offers the first normatively focused consideration of the place of uncertainty in genomic testing. We have considered how uncertainty is going to arise in genomics and have offered a conceptualization of ‘genomic uncertainty’. We have suggested that genomic testing should not be offered merely as a means to reduce uncertainty; nor should uncertainty necessarily be framed negatively or as something that should always be eradicated. Instead, the process of genomic testing should include an explicit consideration of uncertainty, [16] both before and after testing. This should not involve mere education to reduce uncertainty, but encompass a richer engagement involving appraisal, adaptation and complex communication. We have also suggested several ethical concepts that can guide how uncertainty is handled: resilience, welfare, autonomy and solidarity.

For health professionals working in genomics, none of what we have suggested will require significant changes to practice. Rather, we offer this critique as a means to ensure that genomics does not become too focused on uncertainty reduction or eradication. Removing some kinds of uncertainty can introduce other kinds; and uncertainty can sometimes even be valuable or positive [22, 36]. It is encouraging to see counselors already publishing about their experiences in handling uncertainty, including the need to account for it explicitly [15, 16, 27]. Overall, the role of clinicians and counselors is to facilitate those receiving genomic information to come to terms with the certainties and uncertainties that arise during and after the testing process [38].

## Abbreviations

BRCA1: Breast Cancer Genes 1
BRCA2: Breast Cancer Genes 2

## Footnotes

1 A similar claim has been made by Hall et al. [39], namely that the consent process should include discussions of the possibility that results might be ‘inconclusive’ and that this should be regarded as part of routine practice. We also note that genomic testing, whether somatic or germ-line, can lead to greater certainty too.

2 This conceptualisation has been developed in collaboration with Dr Anthony Wrigley.

3 As Babrow et al. [14] note, uncertainty can relate to the status of a certain piece of information, or the ordering of several pieces of information, or how information is synthesized. The information can also have properties such as being incomplete or ambiguous.

4 Babrow et al. term this aspect of uncertainty: ‘complexity’, which Han et al. also use as a descriptor. Babrow et al. use this term more broadly, describing it as the “complexity of whatever is at issue”, whereas Han et al. use it to describe information. Therefore, we have avoided using the same term twice.

5 Note that Han et al. seem to refer to probability as it relates to a source of uncertainty in the test itself; whereas Babrow et al. discuss probability as it is experienced by individuals.

6 It is worth noting that Taber et al. (2015, cited at Reference [10]) found overall low prevalence of this kind of attitude. However, many early adopters of genomic testing are healthy individuals and their attitudes will not necessarily be indicative of those of patients who live with (or have a family history of) a genetic condition.
